# Multidrug-resistant *Escherichia coli* causing diarrhea in yak calves on the Qinghai-Tibet Plateau: phenotypic characterization, whole-genome sequencing, and pathogenicity analysis

**DOI:** 10.14202/vetworld.2026.948-963

**Published:** 2026-03-12

**Authors:** Qian Chen, Di Wu, Zhen Yang, Chang Sun, Shulin Tang, Changjiang Chen, Bin Wei, Qing Liu, Pengxia Bai, Hongjuan Zhang, Shengyi Wang, Baocheng Hao

**Affiliations:** 1 Key Laboratory of New Animal Drug Project, Gansu Province, China; Key Laboratory of Veterinary Pharmaceutical Development, Ministry of Agriculture and Rural Affairs, Lanzhou Institute of Husbandry and Pharmaceutical Sciences of Chinese Academy of Agriculture Sciences, Lanzhou 730050, China; 2 Animal Husbandry and Veterinary Station of Huangyuan County, Xining, 812100, China; 3 Qinghai College of Animal Husbandry and Veterinary Technology, Xining, 812100, China

**Keywords:** antimicrobial resistance, diarrhea, *Escherichia coli*, multidrug-resistant, pathogenicity, Qinghai-Tibet Plateau, whole-genome sequencing, yak calves

## Abstract

**Background and Aim:**

Calf diarrhea represents a major threat to yak (*Bos grunniens*) husbandry on the Qinghai-Tibet Plateau, where extreme environmental conditions (high altitude, low oxygen, cold temperatures) and irregular antibiotic use may accelerate the emergence of multidrug-resistant (MDR) bacterial pathogens. This study aimed to isolate and identify the predominant bacterial agents responsible for diarrhea in yak calves, determine their antimicrobial resistance profiles, and investigate the genomic features and pathogenicity of the most resistant strain to provide evidence-based guidance for prevention and control.

**Materials and Methods:**

Rectal swabs were collected from 12 naturally diarrheic yak calves across four geographically distinct farms in Huangyuan County, Qinghai Province, during the peak season (June–July). Bacterial isolates were obtained through enrichment in Luria-Bertani broth followed by plating on Luria-Bertani agar, and identified by Gram staining, *16S rRNA* gene amplification (primers 27F/1492R), and Sanger sequencing with BLAST comparison (>99.5% identity). Antimicrobial susceptibility was assessed using the Kirby-Bauer disk diffusion method with 17 antibiotics representing eight classes, interpreted according to Clinical and Laboratory Standards Institute VET08-Ed4 breakpoints. The most MDR isolate (HYCQ01) underwent whole-genome sequencing (WGS) on the Oxford Nanopore Technologies MinION platform. Genome assembly quality was evaluated with BUSCO v5.4.7; virulence factors and antibiotic resistance genes were annotated against the Virulence Factor Database and Comprehensive Antibiotic Resistance Database, respectively (BLASTP, e-value ≤ 1e-5, identity ≥ 40%, length ≥ 50 bp). Pathogenicity was tested in 20 male C57BL/6 mice (7–8 weeks, 20 ± 2 g) via intraperitoneal injection of 1.0 × 10^8^ colony-forming units/mL bacterial suspension (100 μl/kg); survival was monitored, and organ histopathology (heart, jejunum, kidneys, liver, lungs, spleen) was examined after hematoxylin-eosin staining. All animal procedures were approved by the Experimental Animal Ethics Committee (No. 2024-030). Data were analyzed using GraphPad Prism 10.1.2 with one-way analysis of variance.

**Results:**

Eight *Escherichia coli* strains were isolated from the 12 samples and confirmed by *16S rRNA* sequencing. All isolates displayed MDR phenotypes, showing 100% resistance to penicillin G and clindamycin, 87.5% to sulfafurazole, and 75.0% to erythromycin. WGS of HYCQ01 revealed 32 resistance classes and 152 resistance genes, consistent with its phenotype (including β-lactamases, macrolide-lincosamide resistance determinants, and tetracycline efflux pumps). Virulence genes included Type III secretion system components, alginate biofilm regulators, iron acquisition systems (*pvdE*), and hemolysins (*rck*). Phylogenetically, HYCQ01 clustered near enterotoxigenic *E. coli* O139:H28 but exhibited a hybrid profile combining animal-associated colonization factors (F17, CFA/I) with atypical extraintestinal traits. In the mouse model, HYCQ01 induced 100% mortality within 27 h post-challenge (p < 0.0001) and caused severe histopathological damage in spleen and jejunum, indicating strong systemic invasiveness.

**Conclusion:**

MDR *E. coli*, exemplified by the hybrid strain HYCQ01, predominates as a causative agent of yak calf diarrhea on the Qinghai-Tibet Plateau, shaped by local ecological pressures and horizontal gene transfer. These results highlight the urgent need for region-specific antimicrobial resistance surveillance, rational antibiotic stewardship, and exploration of non-antibiotic alternatives (probiotics, plant-derived antimicrobials) within a One Health framework. WGS data are deposited at NCBI under BioProject PRJNA1289237.

## INTRODUCTION

Yaks (Bos grunniens) are domesticated mammals unique to the Qinghai-Tibet Plateau, inhabiting alpine regions at elevations of 2,000–6,000 m. They maintain the ecological balance of high altitude pastoral ecosystems while serving as vital economic animals for local communities [1]. Qinghai is the world’s primary yak breeding region, with an annual stock of 5.18 million head, accounting for 38% of China’s total yak population. However, high calf mortality severely constrains the sustainable development of the industry, with diarrhea being the primary cause of illness and death [2–4]. Bacterial pathogens, such as diarrheagenic *Escherichia coli*, *Salmonella* spp., and *Clostridium perfringens*, play pivotal roles in this process. In clinical practice, antibiotics remain the core strategy for controlling such bacterial infections, with their efficacy directly impacting economic losses and disease control outcomes.

However, antimicrobial resistance, particularly multidrug resistance in *E. coli*, has demonstrated multiple adverse consequences and has become a serious global public health issue [5, 6]. Numerous domestic and international studies have focused on testing the susceptibility of multidrug-resistant (MDR) *E. coli* to different antibiotics and characterizing their resistance genes [7]. The Qinghai-Tibet Plateau lies at an average elevation of approximately 4,000 m. Most areas experience an annual average temperature below 5°C, with an average annual oxygen content of approximately 20%. Unlike typical breeding environments, the Qinghai-Tibet Plateau’s extreme geographical and ecological features, high altitude, low oxygen, cold temperatures, and a relatively closed nomadic production system, collectively shape a unique microbial evolutionary pressure environment. More critically, antibiotic access and usage patterns may exhibit significant regional characteristics, such as reliance on a limited number of readily available drugs or non-standardized medication practices, because of the fragmented veterinary service network in pastoral areas. The convergence of this “unique ecological environment” and “specialized antibiotic selection pressure” is highly likely to foster an *E. coli* resistance profile specific to the plateau region. It could even render local strains a reservoir and source for the dissemination of novel or composite resistance genes [8, 9].

Regrettably, the current understanding of this critical interface remains incomplete: What specific resistance phenotypes and genotypes do primary pathogenic bacteria causing calf yak diarrhea exhibit when faced with survival challenges in the extreme high altitude environment? Drug-resistant bacteria and their genes can transmit between animals, the environment, and humans, threatening public health security in frontier pastoral communities and beyond [10, 11].

This study isolated primary pathogenic bacteria from calf yak diarrhea samples collected from typical pastoral areas on the Qinghai-Tibet Plateau. The obtained resistance profiles were comprehensively analyzed, and whole-genome sequencing (WGS) was employed to analyze the genomic characteristics. This study aimed to identify the primary pathogenic bacteria causing diarrhea in local calf yaks, their antimicrobial resistance characteristics, and pathogenicity. It provides a foundational reference for establishing scientifically sound clinical drug use strategies and a regional antimicrobial resistance control system.

## MATERIALS AND METHODS

### Ethical approval

Twenty male C57BL/6 mice, 7–8 weeks old and weighing 20 ± 2 g, were purchased from the Lanzhou Veterinary Research Institute, Chinese Academy of Agricultural Sciences, Lanzhou, China. All mice were maintained on a standard diet and water ad libitum in a temperature- and humidity-controlled environment with a 12 h light/dark cycle. The housing temperature was kept constant at 22°C throughout the experiments. All animal procedures were approved by the Experimental Animal Ethics Committee of Lanzhou Institute of Animal Husbandry and Pharmaceutical Sciences, Chinese Academy of Agricultural Sciences (Ethics license No. 2024-030).

### Sample collection

Huangyuan County, Qinghai Province, hosts a large yak population and widespread pastoral farming. During the peak diarrhea season (June–July), one large yak farm (herd size > 2 000 yaks) was selected from each of four geographically distinct zones (northeast, northwest, southeast, southwest) within the county. Three rectal swab samples were collected from naturally diarrheic yak calves on each farm, yielding a total of 12 samples. All sampled calves were naturally suckled and grazed with their dams on natural pastures. No calves had received antibiotics or antidiarrheal agents prior to sampling. Swabs were immediately placed in sterile transport medium, transported in a –20°C car refrigerator, and transferred to –70°C storage within 48 h for subsequent processing.

### Bacterial isolation and culture

All procedures involving live bacteria were performed in a Biosafety Level 2 laboratory in accordance with protocols for handling MDR pathogens. Equipment and waste were autoclaved before disposal.

Rectal swabs were homogenized in sterile saline inside a biological safety cabinet. For primary enrichment, 100 μL of the suspension was inoculated into non-selective Luria-Bertani (LB) broth (Oxoid, Basingstoke, UK) and incubated aerobically at 37°C for 12 h. To obtain single colonies, 10 μL of the enriched broth was streaked in duplicate onto non-selective LB agar plates (Oxoid) and incubated at 37°C for 18–24 h.

### Phenotypic and microscopic identification

From each sample, three presumptive *Escherichia coli* colonies (selected based on typical morphology and, where applicable, metallic sheen on preliminary eosin methylene blue (EMB) agar screening) were subjected to Gram staining. Colonies showing Gram-negative, rod-shaped morphology were purified by repeated subculture on LB agar.

### Molecular identification

Genomic DNA was extracted from pure cultures using the TaKaRa MiniBEST Bacterial Genomic DNA Extraction Kit (TaKaRa Bio, Dalian, China). DNA concentration and purity (A260/A280 ratio) were determined using a NanoDrop spectrophotometer. The nearly full-length *16S rRNA* gene was amplified using universal primers 27F and 1492R. Polymerase chain reaction was performed in 50 μL reactions containing 25 μL polymerase chain reaction (PCR) Premix, 0.5 μM of each primer, and approximately 5 ng of high-quality DNA template. Thermal cycling conditions were as follows: initial denaturation at 94°C for 5 min; 35 cycles of 94°C for 30 s, 55°C for 30 s, and 72°C for 90 s; final extension at 72°C for 10 min.

PCR products were purified by 1% agarose gel electrophoresis. Sanger sequencing was performed by Sangon Biotech (Shanghai, China). Sequencing reads with Phred quality scores (Q) > 20 were retained for assembly. For phylogenetic analysis, sequences were compared against the NCBI nr database via BLAST, retaining hits with >99.5% identity [12]. The top 30 matches were aligned using MAFFT. A maximum-likelihood phylogenetic tree was constructed with IQ-TREE v2.3.4 (best-fit model determined automatically), with branch support assessed from 1 000 bootstrap replicates. The tree was visualized using FigTree v1.4.4.

### Antimicrobial susceptibility testing

Pathogenic *E. coli* from animal sources in China commonly exhibit high resistance to sulfonamides, tetracyclines, quinolones, β-lactams, lincosamides, and aminoglycosides, while retaining sensitivity to polypeptides, nitrofurans, and fosfomycin [13]. The antibiotics most frequently used on the sampled yak farms included macrolides, β-lactams, tetracyclines, and lincosamides. Accordingly, 17 antimicrobial susceptibility test disks (Liofilchem, Roseto degli Abruzzi, Italy) were selected: penicillin G (PEN, 10 IU, LOT: 021122080), amoxicillin (AMX, 25 μg, LOT: 100322093), ampicillin (AMP, 10 μg, LOT: 051223127), cefotaxime (CTX, 40 μg, LOT: 071423021), cefoxitin (FOX, 30 μg, LOT: 082622013), gentamicin (GEN, 120 μg, LOT: 021422079), kanamycin (KAN, 30 μg, LOT: 120922035), streptomycin (STR, 300 μg, LOT: 102622125), neomycin (NEO, 30 μg, LOT: 120423060), erythromycin (ERY, 15 μg, LOT: 092823080), tetracycline (TCY, 30 μg, LOT: 101422125), ciprofloxacin (CIP, 5 μg, LOT: 072523085), ofloxacin (OFX, 5 μg, LOT: 010324089), norfloxacin (NOR, 10 μg, LOT: 121222076), clindamycin (LIN, 10 μg, LOT: 051322043), trimethoprim-sulfamethoxazole (STX, 25 μg, LOT: 100422119), and sulfafurazole (SUL, 300 μg, LOT: 020722103). All tests were performed in triplicate.

Quality control was conducted using the standard *E. coli* strain ATCC 25922. Results were interpreted according to Clinical and Laboratory Standards Institute (CLSI) VET08-Ed4 breakpoints and classified as susceptible (S), intermediate (I), or resistant (R).

### WGS

The most MDR isolate (designated HYCQ01) was selected for WGS based on the antimicrobial susceptibility results. Sequencing was performed by Sangon Biotech Co., Ltd. (Shanghai, China) using the Oxford Nanopore Technologies MinION platform [14]. The purified strain was cultured in LB broth at 37 °C for 12 h. Bacterial cells from 10 mL of culture were harvested, inactivated by incubation at 60 °C for 30 min in a water bath, and processed for sequencing.

Genome assembly completeness was evaluated using BUSCO v5.4.7 with the OrthoDB dataset. Genomic features of HYCQ01, including GC content, sequencing depth, gene content, and clusters of orthologous groups functional categories, were visualized using Circos software.

Functional annotation of protein sequences was performed against the Virulence Factor Database (VFDB) and the Comprehensive Antibiotic Resistance Database (CARD) using BLASTP (e-value ≤1e-5, percent identity ≥40%, alignment length ≥50 bp) to identify putative virulence factors and antibiotic resistance genes, respectively.

Phylogenetic relationships were inferred from a single-copy core gene set and whole-genome SNP loci using the neighbor-joining method. Whole-genome collinearity with comparator strains was analyzed using MUMmer4 and visualized with the R-circlize package.

### Pathogenicity assay

The HYCQ01 strain was purified and cultured in LB broth at 37°C for 12 h. Twenty mice were randomly divided into two groups: control and HYCQ01-infected. Mice in the infected group received an intraperitoneal injection of bacterial suspension (1.0 × 10^8^ CFU/mL) at a dose of 100 μL/kg body weight. Control mice received an equivalent volume of sterile LB broth.

Mice were monitored every 30 min post-injection. Animals showing severe, irreversible signs of debilitation or obvious distress were immediately euthanized humanely, and time of death was recorded.

After euthanasia, tissues (heart, jejunum, kidneys, liver, lungs, spleen) were collected, fixed in 4% paraformaldehyde for 24 h at 4°C, dehydrated, embedded in paraffin, sectioned at 4 μm thickness, and stained with hematoxylin and eosin (H&E). Pathological changes were examined under a light microscope.

### Statistical analysis

Data analysis and figure generation were performed using GraphPad Prism 10.1.2 (GraphPad Software, Inc., San Diego, CA, USA). Results are presented as mean ± standard error of the mean. Group comparisons were conducted using one-way analysis of variance. A p value < 0.05 was considered statistically significant.

## RESULTS

### Strain isolation

**Figure 1:**
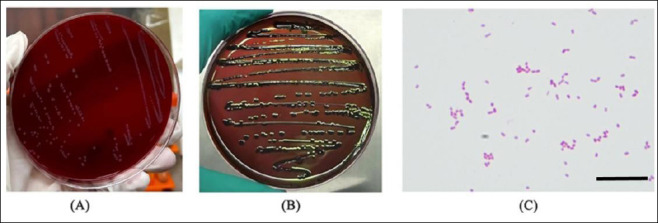
Pathogenic strain cultivation. (A) Isolated strains cultured on blood agar plates showing white, round colonies with smooth surfaces and regular edges. (B) Isolated strains seeded on eosin methylene blue agar plates showing purple-black colonies with a green metallic sheen. (C) Gram staining results showing Gram-negative, rod-shaped bacteria with rounded ends. Scale bar: 10 μm.

There were 8 suspected pathogenic strains isolated from 12 samples. On blood agar plates, the isolated pathogenic strains formed white, round colonies with a smooth surface and regular edges (Figure 1A). They produced purple-black colonies with a green metallic sheen on EMB agar (Figure 1B). Gram staining revealed that the isolated strains were Gram-negative, rod-shaped bacterium with rounded ends (Figure 1C), which were preliminarily identified as *E. coli*.

### Molecular identification

**Figure 2:**
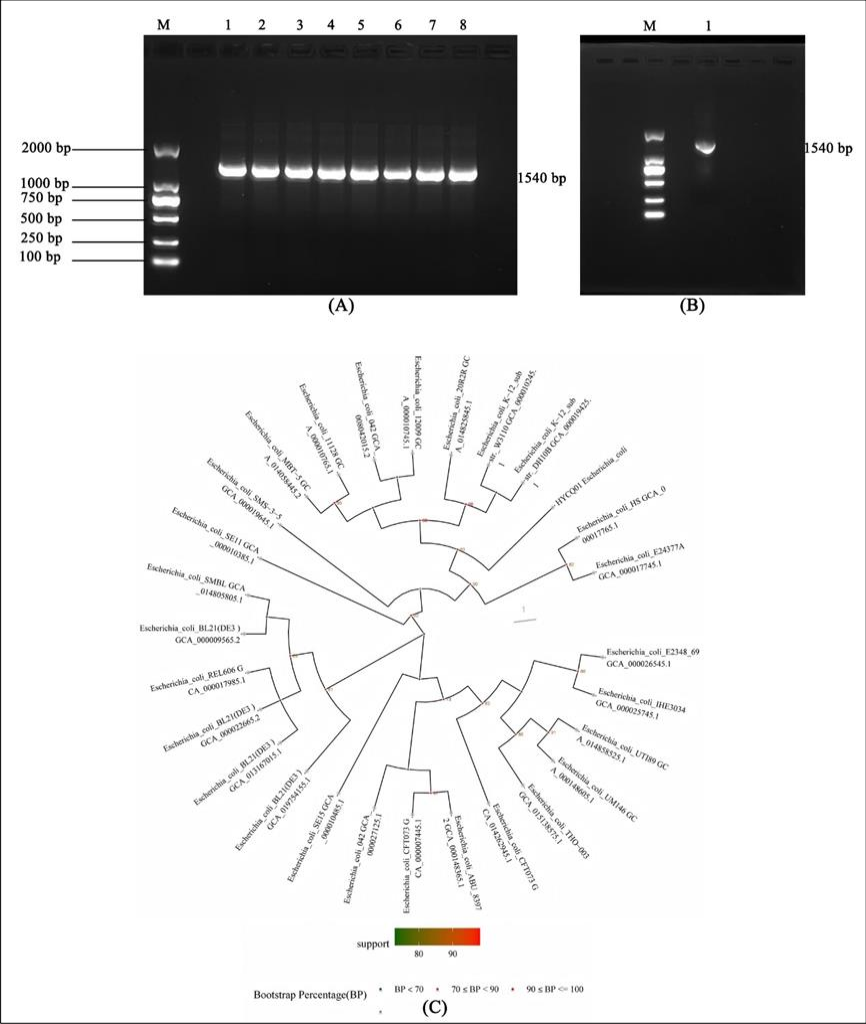
Molecular identification of pathogenic strain HYCQ01. (A) Amplified products of pathogenic bacteria causing yak diarrhea. Lane 1–8: DNA products of the 8 *Escherichia coli* strains; M: DNA marker. (B) Amplified products of HYCQ01. M: DNA marker. (C) BLAST result of the HYCQ01 strain showing closest homology to *E. coli* 20R2R (GCA_014825845.1).

The *16S rRNA* gene amplification products from bacteria were approximately 1540 bp. The band sizes of the eight isolated suspected pathogenic bacteria were consistent with the expected target band size (Figure 2A). The PCR result of HYCQ01 DNA is shown in Figure 2B. Following 16S rRNA PCR amplification and sequencing, the results were compared with the NCBI database by BLAST, confirming the pathogenic isolate as *E. coli*, which is the closest relative to *E. coli* 20R2R GCA_014825845.1 (Figure 2C).

### Antimicrobial susceptibility testing

To explore the antimicrobial susceptibility of HYCQ01, the K-B disk diffusion assay was employed. In total, 17 kinds of antibiotics were selected from 8 types of antibacterial drugs to analyze antibacterial drug susceptibility tests on HYCQ01 (Table 1). All clinical *E. coli* isolates were resistant to more than three antimicrobial drugs. The clinical samples exhibited high levels of resistance to SIZ and ERY (exceeding 75%) and complete resistance (100%) to PEN and LIN. A high level of resistance to β-lactams and macrolides (over 75%) was observed, with complete resistance to lincosamides. In contrast, they were only sensitive to CTX, FOX, GEN, KAN, NEO, SXT, and 4-quinolones, which may be more effective in clinical usage. These results indicate that HYCQ01 is a MDR strain and confirms the existence of its resistance genes.

**Table 1:** Antibiotic susceptibility profiles of eight *Escherichia coli* isolates.

Antibiotic classification	Antibiotic name	Isolate 1	Isolate 2	Isolate 3	Isolate 4	Isolate 5	Isolate 6	Isolate 7	HYCQ01	Resistance rate (%)
β-Lactams	Penicillin G	R	R	R	R	R	R	R	R	100.00
	Amoxicillin	I	R	I	R	R	R	I	R	62.50
	Ampicillin	S	R	I	S	R	I	S	R	37.50
Cephalosporins	Cefotaxime	S	S	S	S	S	S	S	I	0.00
	Cefoxitin	S	I	S	S	S	S	S	S	0.00
Aminoglycosides	Gentamicin	S	S	S	S	S	S	S	S	0.00
	Kanamycin	S	I	I	S	I	I	S	I	0.00
	Streptomycin	I	S	R	I	R	R	R	R	62.50
	Neomycin	S	I	I	S	I	I	I	I	0.00
Macrolides	Erythromycin	R	R	I	I	R	R	R	R	75.00
Tetracyclines	Tetracycline	S	R	I	I	R	I	R	R	50.00
4-Quinolones	Ciprofloxacin	S	I	S	S	S	S	S	R	12.50
	Ofloxacin	S	S	S	S	S	S	S	R	12.50
	Norfloxacin	S	S	S	S	S	S	S	R	12.50
Lincosamides	Clindamycin	R	R	R	R	R	R	R	R	100.00
Sulfonamides	Trimethoprim-sulfamethoxazole	S	S	S	S	S	S	S	S	0.00
	Sulfafurazole	R	S	R	R	R	R	R	R	87.50

Results were interpreted according to Clinical and Laboratory Standards Institute VET08-Ed4 breakpoints. S = susceptible, I = intermediate, R = resistant. Isolates 1–7 represent the seven other *E. coli* strains; HYCQ01 is the most multidrug-resistant strain selected for further WGS and pathogenicity analysis. Resistance rates are calculated across all eight isolates.

### Genomic characterization

**Figure 3:**
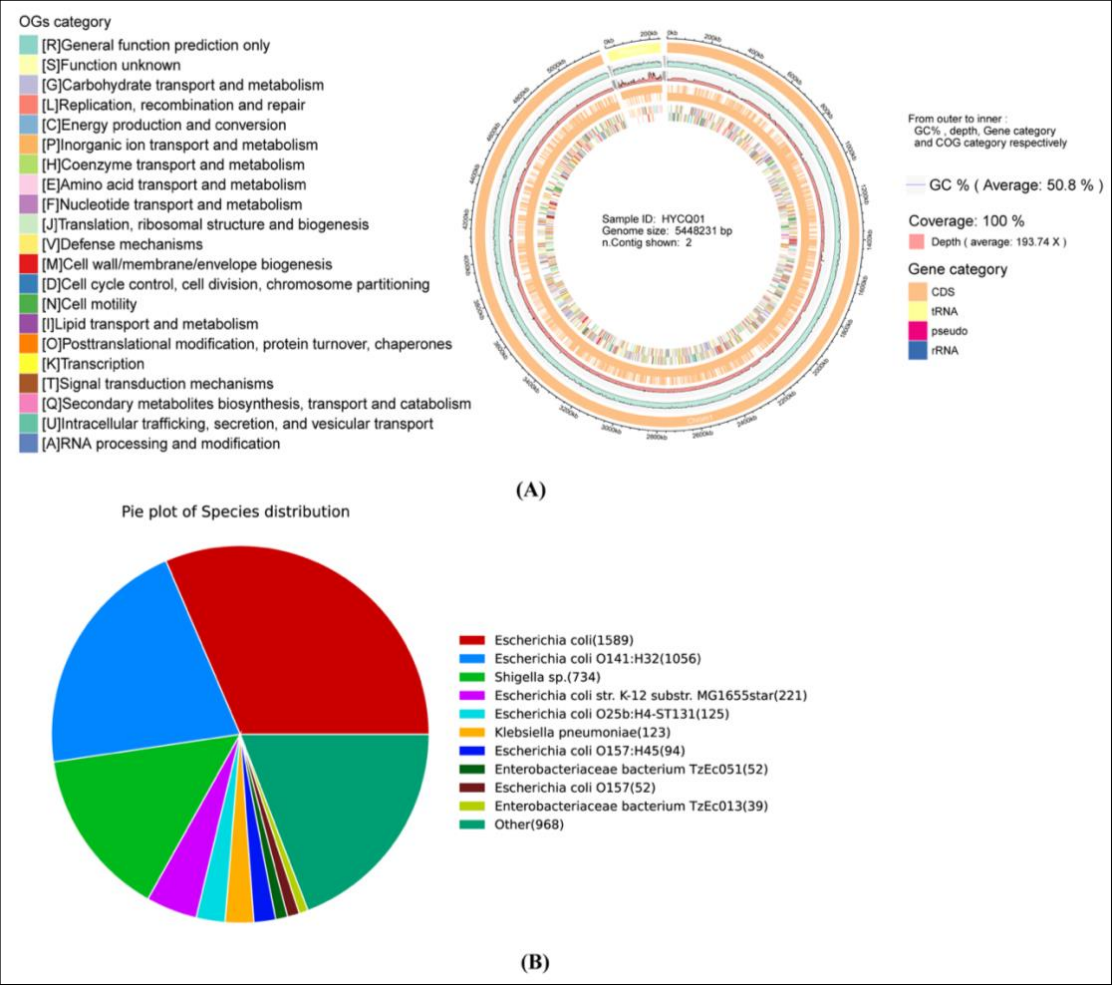
Genomic characterization of HYCQ01. (A) Clusters of orthologous groups category distribution of HYCQ01. (B) Analysis of sequence homology of HYCQ01 against the NR database.

The final assembly had an N50 contig size of 5199568. The completeness and accuracy of the genome assembly revealed 100% completeness of conserved single-copy genes (Supplementary Fig. 1). The complete genome of the HYCQ01 strain was 5,448,231 bp in length, with 50.8% G+C content, depth193.74×, and a gene coverage of 100%, as detailed in Figure 3A. The NR protein database is a non-redundant protein database created and maintained by the NCBI. The database includes comprehensive protein sequence and annotation information along with corresponding species information. Annotation via the NR database revealed that *E. coli* had the highest number of genes showing sequence homology with HYCQ01, with 1589 genes accounting for 31.59% of the total, followed by *E. coli* O141:H32 with 20.99%. (Figure 3B). Consistent with our earlier molecular identification, the results showed that HYCQ01 is the closest relative to *E. coli*.

Blastp analysis against the VFDB database identified a total of 337 and 635 items in VFDB Set A (the core dataset of VFDB) and Set B (the full dataset of VFDB) of the HYCQ01 strain, respectively. Tables 2 and 3 present a summary of the annotations for some virulence factors.

**Table 2:** Toxicity factor annotation results for Set A.

VFDB_A	GeneID	VFG_Symbol	VF_Info	VF_Name
VFG000007	Chrom1_002687, Chrom1_003192	fimB	Chaperone protein	Fimbriae
VFG000033	Chrom1_001763, Chrom1_003452	bplF	Lipopolysaccharide biosynthesis protein	LPS
VFG000035	Chrom1_003447	bplD	UDP-N-acetylglucosamine 2-epimerase	LPS
VFG000044	Chrom1_002472	bscR	*Bordetella* Bsc Type III secretion system protein	TTSS
VFG000048	Chrom1_003397	bscN	ATP synthesis in Type III secretion system	TTSS
VFG000077	Chrom1_004704	clpP	ATP-dependent Clp protease proteolytic subunit	ClpP
VFG000106	Chrom1_002611	acfD	Accessory colonization factor	ACF
VFG000116	Chrom1_002283	algB	Two-component response regulator AlgB	Alginate
VFG000118	Chrom1_003670	algQ	Alginate regulatory protein (AlgQ)	Alginate
VFG000119	Chrom1_001933, Chrom1_001642	algR	Alginate biosynthesis regulatory protein	Alginate
VFG000121	Chrom1_002124	algU	Alginate biosynthesis protein AlgZ/FimS	Alginate
VFG000122	Chrom1_003448	algD	GDP-mannose 6-dehydrogenase AlgD	Alginate
VFG000139	Chrom1_003284	waaG	B-band O-ant polymerase	LPS
VFG000140	Chrom1_003283	waaP	UDP-glucose:(heptosyl) LPS α-1,3-glucosyltransferase WaaG	LPS
VFG000142	Chrom1_003275	waaC	3-deoxy-D-manno-octulosonic-acid transferase	LPS

ACF = Accessory colonization factor, AlgB = Alginate response regulator AlgB, AlgD = GDP-mannose 6-dehydrogenase, AlgQ = Alginate regulatory protein, AlgR = Alginate biosynthesis regulatory protein, AlgU = Alginate biosynthesis regulatory protein AlgZ/FimS, ClpP = ATP-dependent Clp protease proteolytic subunit, LPS = Lipopolysaccharide, TTSS = Type III secretion system.

**Table 3:** Toxicity factor annotation results for Set B.

VFDB_B	GeneID	VFG_Symbol	VF_Info	VF_Name
VFG000035	Chrom1_003447	bplD	UDP-N-acetylglucosamine 2-epimerase	LPS
VFG000077	Chrom1_004704	clpP	ATP-dependent Clp protease proteolytic subunit	ClpP
VFG000079	Chrom1_002145	clpC	Endopeptidase Clp ATP-binding chain C	ClpC
VFG000106	Chrom1_002611	acfD	Accessory colonization factor	ACF
VFG000121	Chrom1_002124	algU	Alginate biosynthesis protein AlgZ/FimS	Alginate
VFG000139	Chrom1_003284	waaG	B-band O-ant polymerase	LPS
VFG000140	Chrom1_003283	waaP	UDP-glucose:(heptosyl) LPS α-1,3-glucosyltransferase WaaG	LPS
VFG000142	Chrom1_003275	waaC	3-deoxy-D-manno-octulosonic-acid transferase	LPS
VFG000160	Chrom1_004589	pvdE	Pyoverdine biosynthesis protein PvdE	Pyoverdine
VFG000177	Chrom1_002601	xcpW	General secretion pathway protein J	XCP secretion system
VFG000313	Chrom1_002399	gluP	Glucose/galactose transporter	LPS
VFG000320	Chrom1_003287	kdtB	Lipopolysaccharide core biosynthesis protein	LPS
VFG000344	Chrom1_000855, Chrom1_005057, Chrom1_000543	hitC	Iron (III) ABC transporter ATP-binding protein	HitABC
VFG000442	Chrom1_005063, Chrom1_000723	rck	Resistance to complement killing	Rck
VFG000449	Chrom1_004801	fimZ	Fimbrial protein Z	Type 1 fimbriae

ACF = Accessory colonization factor, Alginate = Alginate biosynthesis protein AlgZ/FimS, ClpP = ATP-dependent Clp protease proteolytic subunit, ClpC = Endopeptidase Clp ATP-binding chain C, HitABC = Iron (III) ABC transporter system, LPS = Lipopolysaccharide, Pyoverdine = Pyoverdine biosynthesis system, Rck = Resistance to complement killing protein, XCP secretion system = General secretion pathway protein system, Type 1 fimbriae = Fimbrial protein Z.

Blastp analysis against the CARD identified 247 ARO terms (Table 4). Comprehensive analysis of the antibiotic resistance database revealed that the HYCQ01 strain harbored 32 types of antibiotic resistance, including macrolide, TCY, Cephalosporin, Carbapenem, Lincosamide, Penem, Sulfonamide, β-lactam, and vancomycin antibiotics. The isolate HYCQ01 harbored 152 antibiotic resistance genes, including 10 TCY resistance genes, 16 vancomycin resistance genes, 5 macrolide resistance genes, such as Erm (34), emrA, and emrB, β-lactam resistance genes Omp A and Omp K37, a sulfonamide resistance gene sul-4, the AMP resistance gene Tem-1, which was a primary cause of AMP resistance in *E. coli*, and the colistin resistance gene mcr-3.41. Cephalosporin *AmpC* resistance gene was not detected.

**Table 4:** Annotated results of the drug resistance function.

CARD	GeneID	ARO name	Gene family	Resistance mechanism
ARO:3000024	Chrom1_003084, Chrom1_003215, Chrom1_002910	patA	ATP-binding cassette antibiotic efflux pump	Antibiotic efflux
ARO:3000025	Chrom1_003384, Chrom1_002251, Chrom1_004631, Chrom1_002842	patB	ABC antibiotic efflux pump	Antibiotic efflux
ARO:3000027	Chrom1_002259	emrA	Major facilitator superfamily antibiotic efflux pump	Antibiotic efflux
ARO:3000074	Chrom1_002260	emrB	MFS antibiotic efflux pump	Antibiotic efflux
ARO:3000167	Plasmid1_005351	tet(C)	MFS antibiotic efflux pump	Antibiotic efflux
ARO:3000191	Chrom1_002120	tet(Q)	Tetracycline-resistant ribosomal protection protein	Antibiotic target protection
ARO:3000195	Chrom1_002965	tetB(P)	Tetracycline-resistant ribosomal protection protein	Antibiotic target protection
ARO:3000237	Chrom1_002675	TolC	ABC, MFS, and RND antibiotic efflux pumps	Antibiotic efflux
ARO:3000254	Chrom1_001507, Chrom1_001918	emrY	MFS antibiotic efflux pump	Antibiotic efflux
ARO:3000263	Chrom1_000905	marA	General bacterial porin with reduced permeability to β-lactams; RND antibiotic efflux pump	Antibiotic efflux, reduced permeability to antibiotics
ARO:3000499	Chrom1_005128	AcrE	RND antibiotic efflux pump	Antibiotic efflux
ARO:3000504	Chrom1_004756, Chrom1_002931	golS	RND antibiotic efflux pump	Antibiotic efflux
ARO:3000508	Chrom1_000872, Chrom1_004803, Chrom1_003792, Chrom1_003152	gadX	RND antibiotic efflux pump	Antibiotic efflux
ARO:3000516	Chrom1_002257	emrR	MFS antibiotic efflux pump	Antibiotic efflux
ARO:3000518	Chrom1_002983	CRP	RND antibiotic efflux pump	Antibiotic efflux
ARO:3000535	Chrom1_001647, Chrom1_005129, Chrom1_003111	macB	ABC antibiotic efflux pump	Antibiotic efflux
ARO:3000561	Chrom1_004689	Tet(30)	MFS antibiotic efflux pump	Antibiotic efflux
ARO:3000600	Chrom1_004226	Erm(34)	Erm 23S ribosomal RNA methyltransferase	Antibiotic target alteration
ARO:3000617	Chrom1_004260	mecA	Methicillin-resistant PBP2	Antibiotic target replacement
ARO:3000620	Chrom1_003157, Chrom1_004387	adeL	RND antibiotic efflux pump	Antibiotic efflux
ARO:3000656	Chrom1_004731, Chrom1_002903	AcrS	RND antibiotic efflux pump	Antibiotic efflux
ARO:3000676	Chrom1_000533, Chrom1_002190	H-NS	MFS and RND antibiotic efflux pumps	Antibiotic efflux
ARO:3000774	Chrom1_004818	adeA	RND antibiotic efflux pump	Antibiotic efflux
ARO:3000778	Chrom1_002882	adeG	RND antibiotic efflux pump	Antibiotic efflux
ARO:3000781	Chrom1_002014	adeJ	RND antibiotic efflux pump	Antibiotic efflux
ARO:3000784	Chrom1_004819	cmeB	RND antibiotic efflux pump	Antibiotic efflux
ARO:3000792	Chrom1_001504	mdtA	RND antibiotic efflux pump	Antibiotic efflux
ARO:3000803	Chrom1_002904	MexE	RND antibiotic efflux pump	Antibiotic efflux

Analysis of the gene sequence of the HYCQ01 strain by Blastp revealed 58 ARGs, distributed across 12 types and 47 subtypes. The results are shown in Table 5.

**Table 5:** Results of resistance gene annotation.

SARG	GeneID	Subtype	Type
AAC60780	Chrom1_004222	Fosmidomycin_RosB	Fosmidomycin
AAC75243	Chrom1_001701	Multidrug_Bicyclomycin-Multidrug_Efflux_ Protein_Bcr	Multidrug
AAC76696	Chrom1_003331	Multidrug_EmrD	Multidrug
AB158573.p01 gene	Chrom1_004872	Unclassified_RpsD_(Rama_or_Sud2)	Unclassified
ABB53349	Chrom1_000322	Vancomycin_VanR	Vancomycin
ACB17952	Chrom1_003149	Multidrug_MdtE	Multidrug
ACR66838	Chrom1_004223	Trimethoprim_DfrA21	Trimethoprim
AF024666.2.gene33.p01	Chrom1_001067	Chloramphenicol_Chloramphenicol exporter	Chloramphenicol
AF162694.1.gene4.p01	Chrom1_003732	Vancomycin_VanT	Vancomycin
AF336096.1.gene1.p01	Chrom1_001733, Chrom1_000737	Multidrug_Omp36	Multidrug
AF336097.1.gene1.p01	Chrom1_000362	Multidrug_Omp36	Multidrug
AJ459418.gene.p01	Chrom1_002824	Sulfonamide_Sul3	Sulfonamide
AY082011.1.gene2.p1	Chrom1_001212	Vancomycin_VanS	Vancomycin
AY463797.7.gene28.p01	Plasmid1_005351	Tetracycline_TetA	Tetracycline
BAH64410	Chrom1_001071	Multidrug_Bicyclomycin-Multidrug_Efflux_ Protein_Bcr	Multidrug
CP000034.1.gene3672.p01	Chrom1_003031	Multidrug_OmpR	Multidrug
CP000034.1.gene4477.p01	Chrom1_001334, Chrom1_001920	Unclassified_Bacterial regulatory protein LuxR	Unclassified
CP001138.1.gene4273.p01	Chrom1_003032	Unclassified_Transcriptional regulatory protein CpxR	Unclassified
CP001485.1.gene721.p01	Chrom1_001710	Unclassified_Transcriptional regulatory protein CpxR	Unclassified
CP001581.1.gene3143.p01	Chrom1_002066	Chloramphenicol and florfenicol resistance gene	Chloramphenicol

SARG = Structured antibiotic resistance gene database.

**Figure 4:**
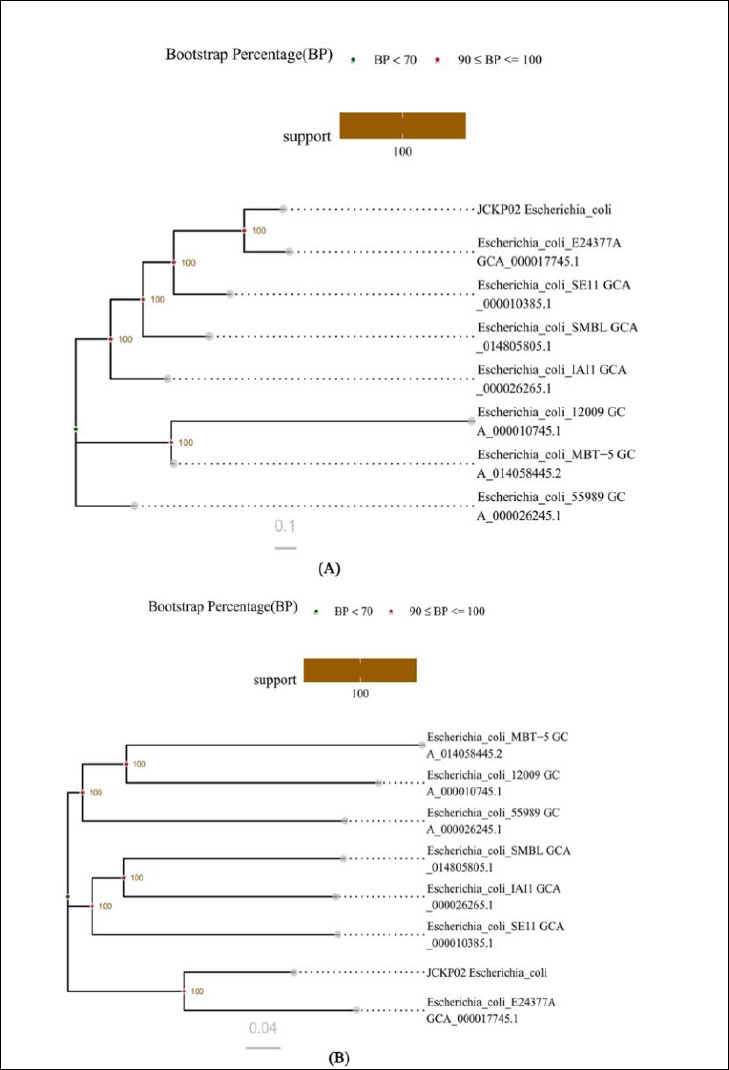
Construction and analysis of the phylogenetic tree. (A) Phylogenetic trees based on single-copy gene sets. (B) Phylogenetic tree based on whole-genome SNP loci.

Based on the results of homologous gene and genome-wide SNP analyses, different phylogenetic trees (core gene tree and SNP tree) were constructed by neighbor-joining clustering with the single-copy gene set of the core gene set and the whole-genome-based SNP loci, respectively. The HYCQ01 strain shared the closest homology with *E. coli* O139:H28(E24377A, ETEC) (Figure 4).

### Pathogenicity assay

**Figure 5:**
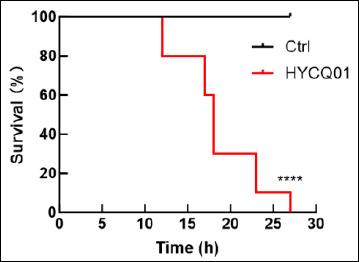
Survival rate of HYCQ01-infected mice. Data are expressed as means ± standard error of the mean of six mice per group. ****p < 0.0001 vs. control group.

The pathogenicity assay was performed in mice to demonstrate the pathogenicity of the HYCQ01 strain. Mice injected with the bacterial suspension exhibited symptoms of lethargy, agitation, and eye closure within 4 h, with progressive mortality commencing at 12 h post-inoculation. All experimental mice died within 27 h. No clinical abnormalities or deaths were observed in the control group (Figure 5). The results demonstrate that HYCQ01 exhibits significant pathogenicity and lethality.

**Figure 6:**
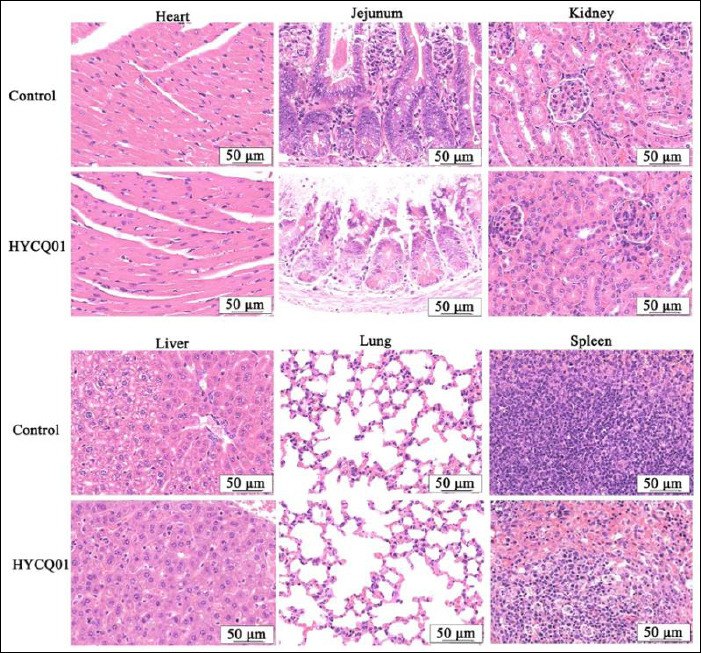
Hematoxylin and eosin staining results in HYCQ01-infected mice. H&E-stained images of the heart, jejunum, kidney, liver, lung, and spleen showing pathological changes. Scale bar: 50 μm.

After all mice had died, we dissected them and found that the jejunum was congested and swollen. Pathological examination of the heart, jejunum, kidneys, liver, lungs, and spleen revealed splenic white pulp atrophy, necrosis, and congestion, whereas mucosal epithelial cell necrosis and villi loss were observed in the jejunum (Figure 6). These data indicate that HYCQ01 is highly toxic and can cause severe tissue damage.

## DISCUSSION

### Primary pathogenic bacterium causing diarrhea in yak calves on the Qinghai-Tibet Plateau

*E. coli* is a prevalent pathogen in livestock, poultry, and associated environments, encompassing over 150 serotypes. In neonatal animals, pathogenic *E. coli* infections frequently induce severe watery diarrhea accompanied by rapid dehydration, resulting in elevated morbidity and mortality rates and substantial economic impacts on animal husbandry [15,16]. Previous reports have indicated that enterotoxigenic *E. coli* represents the predominant *E. coli* pathotype infecting yaks both domestically and internationally, with the highest positivity rate (77.36%) observed in the Tibet region of China [17]. In the present study, *E. coli* was identified as the predominant pathogen isolated from diarrheic yak calves, aligning with findings from prior investigations conducted on the Qinghai-Tibet Plateau and other regions [18–20]. These observations support the inference that pathogenic *E. coli* constitutes the principal etiological agent of diarrhea in yak calves within this geographic area, justifying focused subsequent analyses on this bacterium.

### Adaptation of MDR *E. coli* to the distinctive habitat of the Qinghai-Tibet Plateau

This investigation performed a detailed characterization of MDR *E. coli* isolates from the Qinghai-Tibet Plateau, uncovering a distinctive resistance profile shaped by intricate genetic determinants and pronounced adaptation to the local ecological conditions. The resistance pattern of strain HYCQ01 demonstrated precise correspondence with prevailing environmental selective pressures: it displayed high level resistance to antibiotics frequently employed in local veterinary practice (e.g., β-lactams, macrolides, lincosamides, and tetracyclines), while retaining susceptibility to agents less commonly utilized (e.g., third-generation cephalosporins, quinolones, and most aminoglycosides). This characteristic profile is attributed to the synergistic effects of multiple factors inherent to the unique plateau environment, operating across three principal dimensions.

Historical patterns of antibiotic utilization on the Qinghai-Tibet Plateau serve as the primary driver of this resistance phenotype [21, 22]. Resistance genes harbored by the isolates exhibited strong concordance with observed phenotypic resistance. Resistance to β-lactams was predominantly mediated by the *TEM-1* gene; however, the absence of functional AmpC enzymes (such as CMY-type) likely accounts for the retained susceptibility to CTX and FOX. Elevated resistance to macrolides and lincosamides was principally conferred by the Erm(34) ribosomal methyltransferase in combination with multiple efflux pump genes, reflecting targeted mechanisms against agents routinely applied in livestock production. In agreement with its susceptible phenotype, HYCQ01 lacked prevalent resistance genes associated with quinolones and most aminoglycosides. Moreover, the detection of tetracycline resistance genes (*tet*(C), *tet*(Q)/*tet*B(P)) and sulfonamide resistance gene *sul*3 corroborated selective pressure from corresponding antibiotics in the regional environment. Intriguingly, despite the presence of *sul*3, clinical isolates remained susceptible to SXT, potentially attributable to low expression or incomplete gene functionality precluding phenotypic manifestation, underscoring that genotypic and phenotypic correlations are not invariably perfect. Furthermore, HYCQ01 harbored multiple sequences resembling Gram-positive bacterial resistance genes (e.g., vancomycin-associated genes and *mec*A), which may constitute non-functional remnants or pseudogenes devoid of requisite expression elements in *E. coli*, or alternatively indicate horizontal gene transfer events. This robust genotype-phenotype alignment strongly implicates long-term selective enrichment of the resistance genome under the specific antibiotic exposure regimes characteristic of plateau animal husbandry [23].

Intrinsic tolerance mechanisms elicited by extreme plateau environmental stressors furnish a foundational evolutionary substrate for acquired resistance [24, 25]. A salient feature of this strain is its encoding of a highly complex and stringently regulated multidrug efflux system (e.g., AcrAB-TolC, Mdt series), modulated by global regulators such as *marA* and *emrAB*. These regulatory circuits form central pathways enabling bacterial countermeasures against environmental insults (e.g., oxidative stress and membrane perturbation). The plateau’s intense ultraviolet radiation, pronounced diurnal temperature variations, and low ambient temperatures may persistently stimulate these stress response pathways, thereby constitutively elevating efflux pump expression and conferring baseline tolerance to multiple antimicrobials [26, 27]. This inherent multidrug tolerance augments survival fitness and may diminish the fitness cost associated with acquiring high level resistance mutations or exogenous genes, facilitating their integration and retention within the microbial population [28].

### Pathogenic mechanisms of a hybrid MDR *E. coli* strain from the Qinghai-Tibet Plateau

As a major constituent of the gut microbiota, *E. coli* includes both commensal non-pathogenic strains and virulent pathogenic variants. Pathogenicity assays confirmed that HYCQ01 is not a commensal isolate; its genome encodes functionally active virulence determinants *in vivo*, conferring substantial lethal pathogenic capacity [29]. These empirical findings establish HYCQ01 as a bona fide potential pathogen responsible for calf diarrhea, extending beyond mere genomic associations. Virulence factor annotation revealed that HYCQ01 likely represents a highly invasive hybrid pathogenic *E. coli*. Genes including *fim*B, *fim*Z, and *acf*D contribute to colonization capability [30]. Numerous LPS biosynthesis genes facilitate LPS structural modifications that evade host immune detection. The Type III secretion system (T3SS) enables injection of effector proteins into host cell cytoplasm, constituting a critical determinant of intestinal pathology induced by HYCQ01 in calves and yaks [31]. This mechanism may underlie the jejunoileal lesions observed in challenged mice. As a pivotal virulence factor, T3SS represents a promising target for vaccine and therapeutic development [32]. The iron acquisition gene *pvdE* is typically associated with *Pseudomonas aeruginosa* and encodes a siderophore facilitating iron sequestration from host sources to promote bacterial replication; *rck* encodes a hemolysin that enhances vascular invasion [33, 34]. The coexistence of these elements indicates potential for systemic dissemination by HYCQ01, which may account for the rapid attainment of 100% lethality in poisoned mice. Additionally, HYCQ01 possesses an intact alginate regulatory system and the *pvdE* gene, elements characteristic of *P. aeruginosa*, suggesting acquisition via horizontal gene transfer. Such transfer of resistance and virulence determinants poses a major challenge to effective antimicrobial intervention [35].

Although phylogenetically proximate to enterotoxigenic *E. coli* (ETEC; O139:H28), HYCQ01 cannot be assigned to any of the six canonical enteropathogenic categories owing to the absence of signature toxins or distinctive pathogenic mechanisms [36, 37]. It harbors colonization factors (F17, CFA/I) typical of animal-derived ETEC, yet also exhibits atypical features absent in classical ETEC, including an alginate biofilm system, multiple iron acquisition pathways, and hemolysin production. Thus, HYCQ01 embodies a hybrid pathogen emphasizing colonization and immune evasion, coupled with tissue-damaging capability and extreme MDR. Its emergence signals that, within the Qinghai-Tibet Plateau’s singular ecological niche, drug-resistant bacterial evolution may be progressing toward augmented environmental fitness and pan-resistance.

### Recommendations for prevention and control

The multifaceted attributes of “MDR–environmental adaptability–pathogenic potential” manifested by HYCQ01 carry profound implications for the sustainability of plateau animal husbandry and broader “One Health” public health concerns. Drawing from these results, the following prevention and control strategies are proposed for yak farming on the Qinghai-Tibet Plateau:

Optimize antimicrobial stewardship in clinical practice. Precise antimicrobial selection informed by regional resistance patterns should be prioritized. The HYCQ01 resistance profile mirrors local selective pressures in animal husbandry. Continuous surveillance-based updates to antibiotic utilization guidelines are recommended, with restriction or rotation of agents exhibiting emergent high level resistance (e.g., β-lactams, macrolides) to mitigate source-level selection. Beyond conventional *E. coli* susceptibility testing, advanced diagnostic modalities can improve precision and rapidity; for instance, Cy7-CH3-based near-infrared dual-region luminescent CD kinase probes (CyCDs), employing 3-hydroxytyramine hydrochloride as a surface passivator, enable *in vitro* cell wall staining of resistant bacteria for strain identification and *in vivo* monitoring of host-pathogen interactions to inform timely therapy [38]. Concurrently, adoption of antibiotic alternatives, such as antimicrobial phytochemicals from medicinal plants, probiotic interventions, and immunotherapy, will represent essential future approaches against MDR *E. coli* diarrhea [39–41]. Over recent decades, numerous medicinal plants with broad-spectrum activity against bacterial, viral, and fungal pathogens of human and animal origin have been documented [42, 43].

Prioritize surveillance of mobile genetic elements and vaccine target identification. In parallel with bacterial monitoring, high-risk mobile elements should be tracked [44, 45]. Core colonization factors (e.g., F17 pili) and virulence apparatuses (e.g., T3SS) warrant evaluation as vaccine candidates to provide supplementary control avenues.

Establish a “pasture–environment” antimicrobial resistance genomic surveillance network. Multi-site genomic monitoring encompassing pastures, soil, and aquatic systems is essential to forestall dissemination of this “resistance-virulence reservoir” via hydrological and ecological pathways. Tracking transmission dynamics of key genetic markers, assessing spillover risks to environmental and human compartments, and implementing early warning systems are critical.

### Limitations

Yak husbandry in Huangyuan County, Qinghai Province, predominantly follows nomadic patterns with extensive herd mobility, impeding systematic collection of extensive clinical specimens. Despite maximal implementation of spatially distributed sampling, the constrained sample volume limits extrapolation of findings. Future efforts should incrementally increase sample sizes through prolonged sampling durations, collaboration with herder networks, and adoption of longitudinal dynamic tracking. Long-term cohort monitoring will enable a more robust elucidation of pathogen population dynamics, antimicrobial resistance evolution, and transmission patterns in diarrheic yak calves within this region.

## CONCLUSION

This investigation identified MDR *E. coli* as the predominant etiological agent of diarrhea in yak calves on the Qinghai-Tibet Plateau, with eight *E. coli* strains isolated from 12 rectal swab samples collected from diarrheic calves across four farms in Huangyuan County, Qinghai Province. All isolates exhibited MDR phenotypes, demonstrating 100% resistance to penicillin G and clindamycin, 87.5% to sulfafurazole, and 75.0% to erythromycin. The most resistant strain, HYCQ01, underwent WGS, revealing a genome size of 5,448,231 bp with 50.8% G+C content, encompassing 32 resistance classes and 152 resistance genes, including β-lactamases (*TEM-1*), macrolide-lincosamide determinants (erm(34)), and tetracycline efflux pumps (tet(C), tet(Q)/tetB(P)). Virulence factor annotation identified 337 and 635 factors in Virulence Factor Database sets A and B, respectively, featuring Type III secretion system components, alginate biofilm regulators, iron acquisition systems (pvdE), and hemolysins (rck). Phylogenetically, HYCQ01 clustered near enterotoxigenic *E. coli* O139:H28 but displayed a hybrid profile with animal-associated colonization factors (F17, CFA/I) and extraintestinal traits. In vivo pathogenicity assays in C57BL/6 mice demonstrated 100% mortality within 27 h post-intraperitoneal challenge (*p* < 0.0001), accompanied by severe histopathological lesions in the spleen and jejunum, underscoring systemic invasiveness.

The emergence of hybrid MDR *E. coli* strains like HYCQ01, shaped by the plateau’s extreme environmental stressors and selective antibiotic pressures, poses significant threats to yak husbandry sustainability and public health under a One Health framework. These findings advocate for region-specific antimicrobial stewardship, including restriction of high resistance drugs (e.g., β-lactams, macrolides) and adoption of alternatives such as probiotic interventions and plant-derived antimicrobials [39–43]. Enhanced surveillance of mobile genetic elements and virulence factors could inform vaccine development targeting core systems like Type III secretion system and F17 pili. Establishing a genomic early warning network across pastures, soil, and water bodies would mitigate dissemination risks, ultimately reducing economic losses in pastoral communities and curbing spillover to human populations [10, 11].

This research integrates phenotypic, genomic, and in vivo pathogenicity analyses to provide a comprehensive characterization of MDR *E. coli* in an understudied high altitude ecosystem. The use of WGS on the Oxford Nanopore Technologies MinION platform enabled high-resolution annotation of resistance and virulence genes, while the mouse model offered empirical validation of pathogenic potential. Sampling from geographically dispersed farms during peak diarrhea season enhanced ecological relevance, and adherence to Clinical and Laboratory Standards Institute guidelines ensured robust antimicrobial susceptibility data.

The nomadic nature of yak farming in Huangyuan County restricted sample collection to 12 specimens, potentially limiting generalizability across the broader Qinghai-Tibet Plateau. Reliance on a single MDR strain (HYCQ01) for in-depth genomic and pathogenicity studies may not fully capture intraspecies variability. Additionally, the in vivo model employed mice, which, while informative, may not perfectly recapitulate yak-specific host-pathogen dynamics.

Future investigations should expand sample sizes through longitudinal tracking and herder collaborations to elucidate pathogen population structures and transmission dynamics. Comparative genomics across plateau regions could identify additional hybrid strains and horizontal gene transfer hotspots. Exploring non-antibiotic interventions, such as Cy7-CH3-based probes for rapid resistance detection [38] or berberine-probiotic combinations [42], warrants evaluation in field trials. Integrating metagenomics to assess environmental reservoirs of resistance genes would further inform One Health strategies [44, 45].

In summary, MDR *E. coli* exemplifies an adaptive pathogen thriving in the Qinghai-Tibet Plateau’s unique habitat, driven by ecological pressures and gene exchange events. These insights underscore the imperative for targeted interventions to safeguard animal health, economic viability, and public safety, highlighting the plateau as a critical frontier in global antimicrobial resistance research.

## DATA AVAILABILITY

The data used to support the findings of this study are included within the manuscript. Meanwhile, the WGS and assembly results are deposited at the National Center for Biotechnology Information under accession number: PRJNA1289237.

## AUTHORS’ CONTRIBUTIONS

QC and BCH: Conceived and designed the study. CJC, BW, QL, and PXB: Sample collection, library preparation, and detection work. QC and DW: Designed the computational framework, analysis, and performed data processing, statistical analysis, interpretation of results and drafted the manuscript. SYW and BCH: Supervised the study, interpreted the results, and drafted and revised the manuscript. All authors have read and approved the final version of the manuscript.
